# Hot flushes and quality of life during menopause

**DOI:** 10.1186/1472-6874-9-13

**Published:** 2009-05-18

**Authors:** Riitta Luoto

**Affiliations:** 1 UKK Institute for Health Promotion, Tampere, Finland; 2 National Institute for Health and Welfare, Helsinki, Finland

## Abstract

Menopausal health is important since this stage of life is not to be avoided. A recent article in *BMC Women's Health *from the Estonian Postmenopausal Hormone Therapy trial has concluded that quality of life is not related to hormonal therapy use. The commentary article discusses this finding and considers other factors related to symptoms and quality of life during menopause. Important factors known to affect hot flushes and quality of life are smoking and high body weight. Since both these factors are modifiable, menopause is a suitable area for health promotion. However, evidence concerning lifestyle changes in symptom relief or increase of quality of life is weak. More trials in this area are needed before women may consider non-pharmacological treatment of symptoms as a reliable option for menopausal symptom cure.

## Menopausal symptoms are common

Menopause is not avoided by any woman but frequency and severity of menopausal symptoms vary. A recent article published in *BMC Women's Health *by Veerus *et al *[1] reported that quality of life did not depend on hormonal therapy (HT) use, although HT users reported less hot flushes and sleeping problems. A number of studies have reported the prevalence of menopausal symptoms among mid-aged women. These studies are based on different populations, questions and severity of symptoms. Typical menopausal symptoms are vasomotor symptoms (hot flushes), vaginal dryness [2-4] and sleep disturbances [5]. The probability of other symptoms is higher if the woman has vasomotor symptoms [6,7]. Cross-sectional surveys among women of different ages show that hot flushes are most common soon after menopause, but they occur even after 10 years [4]. According to a Dutch cross-sectional survey, a third of menopausal women have severe vasomotor symptoms and 7% have symptoms even 10 years after the menopause [6,8]. About half of 50-year old and one quarter of 60-year old Swedish women experienced hot flushes [9]. However, not all women consider hot flushes disturbing [6,8-11] and the question of severity remains to be solved.

## Hormone therapy and quality of life

During recent years hormonal therapy (HT) has been introduced as a solution to menopausal symptoms. The Women's Health Initiative's (WHI) study results [12] showed the pros and cons of HT among women aged 65 and over, but HT is known to be an effective relief to hot flushes. **Veerus *et al*'s **study [1] was based on the Estonian Postmenopausal Hormone Therapy (EPHT) trial, which included 1823 women aged 50 to 64 who were followed on average for 3.6 years. HT users reported fewer hot flushes, sweating and sleep problems but did not have better quality of life. The Women's Health Initiative trial reported similar findings to that of Veerus *et al *[13] but in the Heart and Estrogen/Progestin Replacement Study (HERS) trial HT improved quality of life for women with menopausal symptoms [14]. Both HERS and WHI studies had shorter follow-up times than EPHT, which, in addition to cultural factors, explains part of the contradictory result. However, the conclusion is now that quality of life cannot be guaranteed with HT use, it is necessary to consider other issues. It is known that menopausal transition does not intrinsically lead to poor general quality of life (OQL) or satisfaction in life [15]. A crucial factor is presence of vasomotor symptoms, which are clearly related to poor QOL [16]. The result of the Veerus *et al *study [1] was important, since based on these earlier findings it was expected that quality of life would increase if vasomotor symptoms are decreased.

## Lifestyle and menopausal symptoms

Early onset of menopause [17] as well as long perimenopausal phase [18] are risk factors for deteriorated well-being in menopause. Other risk factors for increased experience of hot flushes are smoking and high body mass index (BMI). High BMI (at least 25 kg/m2) has been associated with the risk of any or daily hot flushes in many recent studies [19-22]. All previous studies concluded that associations between hot flushes and BMI were found only among pre- or perimenopausal women, but not among postmenopausal women. Associations between hot flushes and smoking have been shown in many earlier studies [20,23,24].

The quality of life of smokers and persons with high BMI is known to be low and an increase in hot flushes does not alleviate their situation, quite the opposite. There is a clear need for general health promotion and obesity prevention during the menopausal age in order to increase quality of life. Lifestyle changes, such as increasing physical activity, is one alternative to HT. Evidence on reducing cardiovascular disease by physical activity exists especially among postmenopausal women [25,26]. However, evidence on whether physical activity reduces menopausal symptoms is still inconclusive. Earlier studies have suggested physical activity may act as a possible tool for decreasing some menopausal vasomotor symptoms [20,27]. At least maintaining or increasing physical activity in SWAN (Study of Women across Nation) cohort resulted in maintenance or decrease of body weight [28]. Only a few randomized clinical trials on exercise and menopausal symptom reduction have been performed [27,29]. Wilbur *et al *[27] concluded that women in the exercise intervention group had fewer vasomotor symptoms and improved sleep as compared to controls. Aiello *et al *[29] study showed an increase in hot flash severity and decreased risk of memory problems among the intervention group women compared to controls. Both studies were quite small and not all participants had symptoms at baseline. Randomized clinical trials are needed to further address the effect of physical activity for symptoms experienced by midlife women.

## Menopausal health promotion – a future challenge

Menopausal transition may make women more aware of future health risks due to increased symptomatology and help-seeking behaviour. Motivation for health promotion may be further strengthened if women perceive life-style modifications as an alternative, non-pharmacological, way of managing menopausal symptoms. However, more evidence on effectiveness and efficacy of lifestyle changes, especially exercise, on decreasing hot flushes and increasing quality of life is urgently needed. In the future, menopause may act as a window of opportunity for health promotion and life-modifications. Studies from randomized trials such as Veerus *et al *[1] are necessary before taking the next steps in menopausal women's health.

## Authors' contributions

RL originated the idea for the commentary article and is in charge of the article.

## Pre-publication history

The pre-publication history for this paper can be accessed here:



## References

[B1] Veerus P, Fischer K, Hovi S-L, Karro H, Rahu M, Hemminki E (2008). Symptom reporting and quality of life in the Estonian Postmenopausal Hormone Therapy Trial. BMC Women's health.

[B2] Hemminki E, Topo P, Kangas I (1995). Experience and opinions of climacterium by Finnish women. European Journal of Obstetrics & Gynecology and Reproductive Biology.

[B3] Berg JA, Taylor DL (1999). Symptom Experience of Filipino American Midlife Women. Menopause.

[B4] Kronenberg F (1990). Hot Flashes: Epidemiology and Physiology. Ann NY Acad Sci.

[B5] Polo-Kantola P, Saaresranta T, Polo O (2001). Aetiology and treatment of sleep disturbancies during perimenopause and postmenopause. CNSDrugs.

[B6] Oldenhave A, Netelenbos C (1994). Pathogenesis of climacteric complaints: ready for the change?. Lancet.

[B7] Bardel A, Wallander MA, Svarsudd K (2002). Hormone replacement therapy and symptom reporting in menopausal women. A population-based study of 35–65-year-old women in mid-Sweden. Maturitas.

[B8] Oldenhave A, Jaszmann LJB, Haspels AA, Everaerd WT (1993). Impact of climacteric on well-being: a survey based on 5213 women 39 to 60 years old. Am J Obstet Gynecol.

[B9] Rödström K, Bengtsson C, Lissner L, Milsom I, Sundh V, Björkelund C (2002). A longitudinal study of the treatment of hot flushes: the population study of women in Gothenburg during a quarter of a century. Menopause.

[B10] Holte A (1991). Prevalence of climacteric complaints in a representative sample of middle-aged women in Oslo, Norway. J Psychosom Obstet Gynaecol.

[B11] Stearns V, Ullmer L, Lopez JF, Smith J, Isaacs C, Hayes DF (2002). Hot flushes. Lancet.

[B12] Writing group for the Women's Health Initiative investigators (2002). Risks and benefits of the estrogen plus progestin in healthy postmenopausal women: principal results from the Women's health Initiative randomized controlled trial. Journal of the American Medical Association.

[B13] Hays J, Ockene JK, Brunner RL, Kotchen JM, Manson JE, Patterson RE, Aragaki AK, Shumaker SA, Brzyski R, LaCroix AZ, Granek IA, Valanis BG (2003). Women's Health Initiative Investigators. Effects of estrogen plus progestin on health-related quality of life. N Engl J Med.

[B14] Hlatky MA, Boothroyd D, Vittinghoff E, Sharo P, Whooley MA, Hear and Estrogen/Progestin Replacement Study (HERS) Research Group (2002). Quality-of-life and depressive symptoms in postmenopausal women after receiving hormone therapy: results from the Heart and Estrogen/Progestin Replacement Study (HERS) trial. JAMA.

[B15] Avis NE, Assmann SF, Kravitz HM, Ganz PA, Ory M (2004). Quality of life in diverse groups of midlife women: assessing the influence of menopause, health status and psychosocial and demographic factors. Qual Life Res.

[B16] Kumari M, Stafford M, Marmot M (2005). The menopausal transition was associated in a prospective study with decreased health functioning in women who report menopausal symptoms. J Clin Epidemiol.

[B17] Koster A, Eplov LF, Garde K (2002). Anticipations and experiences of menopause in a Danish female general population cohort born in 1936. Arch Women Ment Health.

[B18] Mishra G, Kuh D (2006). Perceived change in quality of life during the menopause. Soc Sci Med.

[B19] den Tonkelaar I, Seidell JC, vanNoord PA (1996). Obesity and fat distribution in relation to hot flashes in Dutch women from the DOM-project. Maturitas.

[B20] Gold EB, Sternfeld B, Kelsey JL, Brown C, Mouton C, Reame N, Salamone L, Stellato R (2000). Relation of demographic and lifestyle factors to symptoms in multi-racial/ethnic population of women 40–55 years of age. American Journal of Epidemiology.

[B21] Whiteman MK, Staropoli CA, Langenberg PW, McCarter RJ, Kjerulff KH, Flaws JA (2003). Smoking, body mass, and hot flashes in midlife women. Obstet Gynecol.

[B22] Riley EH, Inui TS, Kleinman K, Connelly MT (2004). Differential association of modifiable health behaviours with hot flashes in perimenopausal and postmenopausal women. J Gen Intern Med.

[B23] Staropoli CA, Flaws JA, Bush TL, Moulton AW (1998). Predictors of menopausal hot flashes. J Womens Health.

[B24] Avis NE, Crawford SL, McKinlay SM (1997). Psychosocial, behavioural, and health factors related to menopause symptomatology. Womens Health.

[B25] Kushi LH, Fee RM, Folsom Arm Mink PJ, Anderson KE, Sellers TA (1997). Physical activity and mortality in postmenopausal women. JAMA.

[B26] Asikainen T-M, Kukkonen-Harjula K, Miilunpalo S (2004). Exercise for health for early postmenopausal women. Sports Med.

[B27] Wilbur JE, Miller AM, McDevitt J, Wang E, Miller J (2005). Menopausal status, moderate-intensity walking and symptoms in midlife women. Research and theory for nursing practice: an International Journal.

[B28] Sternfeld B, Wang H, Quesenberry CP, Abrams B, Everson-Rose SA, Greendale G, Matthews KA, Torrens JI, Sowers M (2004). Physical activity and changes in weight and waist circumference in midlife women: findings from the study of Women's health Across the Nation. Americal Journal of Epidemiology.

[B29] Aiello EJ, Yasui Y, Tworoger SS, Ulrich CC, Irwin ML, Bowen D, Schwartz RS, Kumai C, Potter JD, McTiernan A (2004). Effect of a yearlong, moderate-intensity exercise intervention on the occurrence and severity of menopause symptoms in postmenopausal women. Menopause.

